# Epidemiology of spinal injuries in the United Arab Emirates

**DOI:** 10.1186/s13017-015-0015-8

**Published:** 2015-05-09

**Authors:** Michal Grivna, Hani O. Eid, Fikri M. Abu-Zidan

**Affiliations:** Institute of Public Health, College of Medicine and Health Sciences, UAE University, Al Ain, United Arab Emirates; Trauma Group, Department of Surgery, College of Medicine and Health Sciences, UAE University, Al Ain, United Arab Emirates

**Keywords:** Spine injury, Fall, Road-traffic crash, Prevention, United Arab Emirates

## Abstract

**Aim:**

To assess the risk factors, mechanism of injury, and clinical outcome of hospitalized patients with spinal injuries in order to recommend preventive measures.

**Methods:**

Patients with spinal injuries admitted to Al Ain Hospital, United Arab Emirates (UAE) for more than 24 h or who died after arrival to the hospital were studied over 3 years. Demography, location and time of injury, affected body regions, hospital and ICU stay, and outcome were analyzed.

**Results:**

239 patients were studied, 90 % were males, and 84 % were in the productive years of 25–54. Majority were from the Indian subcontinent (56 %). Road was the most common location for spinal injury (47 %), followed by work (39 %). The most common mechanism of injury was traffic collisions (48 %) followed by fall from height (39 %) and fall from the same level (9 %). UAE nationals were often injured at road and home compared with non-UAE nationals, who were more injured at work (*p* < 0.0001). Patients falling from the same level were older (*p* = 0.001) and predominantly females (*p* < 0.0001) when compared with other mechanisms. Spinal fractures were more common in the lumbar region (57 %). Eleven patients (5 %) sustained paraplegia and five (4 %) patients died.

**Interpretation:**

Traffic injuries and falls were the leading causes for spinal injuries in the UAE. Expatriate males are at high risk for fall from height, UAE national males for traffic injuries and females for falls at the same level at homes. Prevention should focus on traffic and home injuries for UAE nationals and occupational safety for expatriate workers.

## Introduction

Spinal injury is one of the most devastating injuries having a great impact on patients, their families, and the society [1,2]. It may lead to serious disability when involving the spinal cord with long-term medical complications, including pressure ulcers, autonomic dysreflexia, deep venous thrombosis and pneumonia [3-5]. This significantly impacts rehabilitation and long-term quality of life [4]. People with spinal injury have a high level of distress, depression, anxiety, and suicide attempts because of their lower levels of life satisfaction [6,7]. The costs of spine injuries and their effects on the health care systems are high [8].

United Arab Emirates (UAE), having a predominantly urban population of more than 6 million, is a fast developing country. It is a federation of seven emirates with modern road infrastructure and a high proportion of expatriate workers [9]. Various ethnic groups with sociocultural, religious and educational diversity pose a special challenge for health and safety [10]. In order to propose useful preventive measures, it is necessary to conduct proper epidemiological studies. As there is little information about spine injuries in the Middle East, we aimed to assess the mechanism of injury, severity and outcome of hospitalized spinal injured patients in the UAE in order to give recommendations regarding their prevention.

## Patients and methods

### Ethics statement

The Local Ethics Committee of Al Ain Health District Area approved the study (UAE RECA/02/44). All patients or their care givers signed a consent form for permitting the use of anonymous data for research or audit.

### Setting

Al Ain Hospital is one of the two major hospitals (Al Ain and Tawam Hospitals) in Al Ain City serving a population of about half a million residing in the largest city located in the east of Abu Dhabi Emirate of the UAE [11]. It is a specialized acute care and emergency hospital with 402 beds and more than 35 medical departments and divisions [12]. Around eighty percent of the trauma patients of Al Ain City were treated in Al Ain Hospital during the study period.

### Data collection and scoring

All patients who were admitted to Al Ain Hospital for more than 24 h or who have died after admission following their injury were included in Al Ain Hospital Trauma Registry. Data were collected prospectively from March 2003 to March 2006 on a specially designed hard copy form [13]. A full time Trauma Research Fellow collected data on daily basis on the injured patients and followed them up through their hospital stay.

The data of all patients with spinal injury were retrieved from the registry. The demography of the patients, the mechanism and location of their injury, factors reflecting injury severity and outcome including Glasgow Coma Scale (GCS) on arrival, Injury Severity Score (ISS), Intensive Care Unit (ICU) admission, mortality and neurological deficit were studied. The ISS as a global marker of injury severity was calculated manually using the Abbreviated Injury Scale (AIS) 1998 handbook [14].

### Statistical analysis

Nationality was categorized into two groups – UAE nationals and non-UAE nationals, because previous studies have shown that injury risks for UAE nationals differ from other nationalities [15,16]. Mechanism of injury was categorized into three groups – traffic-related, fall from height, and fall from the same level. Non parametric statistical methods were used in comparing two or three groups because the numbers of subjects in some of the groups were small. Non-parametric statistical methods are advised in this situation because they compare the ranks, normal distribution is not required, and this has a protective effect for the analysis. [17]. Mann–Whitney *U* test was used to compare continuous or ordinal data of two groups while Kruskal–Wallis test was used to compare continuous or ordinal data of three groups. Fisher’s exact test was used to compare categorical data. Analysis was performed using PASW Statistics 21, SPSS Inc, USA.

## Results

There were 239 patients, 215 males (90 %) (male:female ratio was 9:1). The mean (SD) age was 37.5 (12.5) years. Adults in the productive years of 25–54 years were majority (84 %; *n* = 201). Five percent (*n* = 12) were children and youth less than 19 years, and 3 % (*n* = 7) were elderly more than 65 years. Majority were from the Indian subcontinent (55 %; *n* = 132), followed by Arabs (23 %; *n* = 54), UAE nationals (14 %; *n* = 34), and other nationalities 8 % (*n* = 19) (Table 1).

**Table 1:** Hospitalized patients with spine injury by nationality, Al Ain, United Arab Emirates, 2003–2006 (*n* = 239)

	Number	Percent
Pakistan	62	25.9
India	45	18.8
Bangladesh	25	10.5
UAE	34	14.2
Other Arabs	54	22.6
Others	19	7.9
Total	239	100.0

**Fig. 1:**
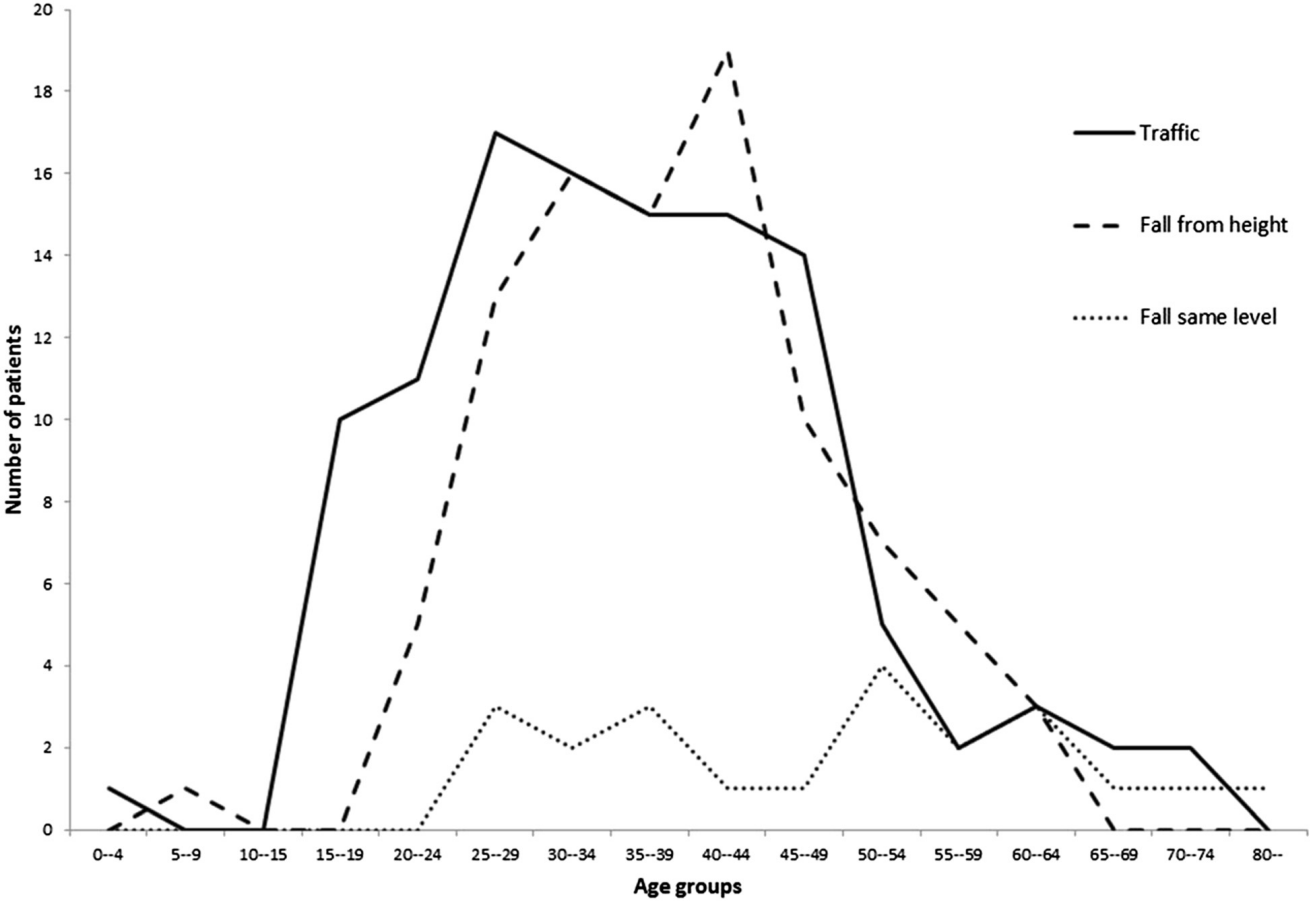
Age distribution of patients with spine injury by mechanism (*n* = 114 traffic; *n* = 94 fall from height; *n* = 22 fall from same level) traffic = solid line; fall from height = interupted line; fall from same level = dotted line

Road was the most common location for spine injury (47 %; *n* = 112), followed by work (39 %; *n* = 93), home (10 %; *n* = 23) and other locations (5 %; *n* = 11). The most common location for injuries for males was road (47 %; *n* = 101) and for females was home (50 %; *n* = 12). UAE nationals were often injured at road and home compared with non-nationals who were injured at work and road (*p* < 0.0001) (Table 2). Traffic-related spine injury was the most common mechanism of injury (48 %; *n* = 114), followed by fall from height (39 %; *n* = 94), fall from same level (9 %; *n* = 21) and other mechanisms, such as falling objects and animal-related injuries (4 %; *n* = 10) (Table 3). Majority of falls fom height occurred at work (85 %; *n* = 80), while majority of falls at the same level occurred at home (68 %; *n* = 15) (*p* < 0.0001) (Table 3). UAE nationals were more injured by traffic and fall from the same level, compared with non-nationals who were more injured by falling from height at work (*p* < 0.0001) (Table 2). Those who had spinal injury at home were significantly older than those who fell from height or were injured by road traffic collisions (*p* = 0.001) (Fig. 1 and Table 3).

**Table 2:** Demographic, location, mechanism, severity, and outcome variables of spine injuries by nationality, Al Ain, UAE (*n* = 239)

Variable		UAE	Non UAE	*p*-value
		(*n* = 34)	(*n* = 203)	
Age (years)		30 (7–80)	35.5 (2–70)	0.2
Gender (male)		26 (76.5 %)	187 (92.1 %)	0.01
Location	Home	10 (29 %)	13 (6.4 %)	<0.0001
	Work	1 (3 %)	99 (48.8 %)	
	Road	22 (65 %)	87 (42.8 %)	
	Other	1 (3 %)	4 (2 %)	
Mechanism	Road traffic injury	23 (67.6 %)	90 (44.3 %)	<0.0001
	Fall from height	3 (8.8 %)	90 (44.3 %)	
	Fall from same level	8 (23.5 %)	13 (6.4 %)	
	Other	0	9 (4.5 %)	
Severity	ICU admission	8 (23.5 %)	21 (10.3 %)	0.044
	Hospital stay (days)	6 (1–21)	8 (1–78)	0.005
	GCS	15 (3–15)	15 (3–15)	0.06
	ISS	5 (4–29)	5 (1–38)	0.06
Outcome	Paraplegia	1 (2.9 %)	10 (4.9 %)	1
	Mortality	2 (5.9 %)	3 (1.5 %)	0.14

*p* = Mann Whitney *U* test or Fisher’s Exact test as appropriate

Data are presented as median (range) or number (%) as appropriate

**Table 3:** Demographic, location, mechanism, severity and outcome variables of spine injuries by mechanims, Al Ain, United Arab Emirates, 2003–2006 (*n* = 239)

Variable		Traffic	Fall from height	Fall at same level	*p*-value
		(*n* = 114)	(*n* = 94)	(*n* = 22)	
Age (years)		35 (2–70)	37 (7–64)	50 (25–80)	0.001
Gender (male)		103 (90.4 %)	90 (95.7 %)	14 (63.6 %)	<0.0001
UAE nationality		23 (20.2 %)	3 (3.2 %)	8 (36.4 %)	<0.0001
Location	Home	0	8 (8.5 %)	15 (68.2 %)	<0.0001
	Work	1 (0.9 %)	80 (85.1 %)	5 (22.7 %)	
	Road	111 (97.4 %)	1 (1.1 %)	0	
	Other	2 (1.8 %)	5 (5.3 %)	2 (9.1 %)	
Severity	ICU admission	24 (21.1 %)	6 (6.4 %)	0	0.001
	Hospital stay (days)	8 (1–77)	17.5 (1–57)	5.5 (1–19)	0.11
	GCS	15 (3–15)	15 (4–15)	15 (15–15)	<0.0001
	ISS	8 (1–38)	4 (1–34)	4 (1–9)	<0.0001
Outcome	Paraplegia	6 (5.3 %)	5 (5.3 %)	0	0.8
	Mortality	5 (4.4 %)	0	0	0.13

*p* = Kruskal–Wallis test or Fisher’s Exact test as appropriate

Data are presented as median (range) or number (%) as appropriate

9 patients with other mechanisms were not included in the analysis

Number may not add to 100 % because of missing data

**Fig. 2:**
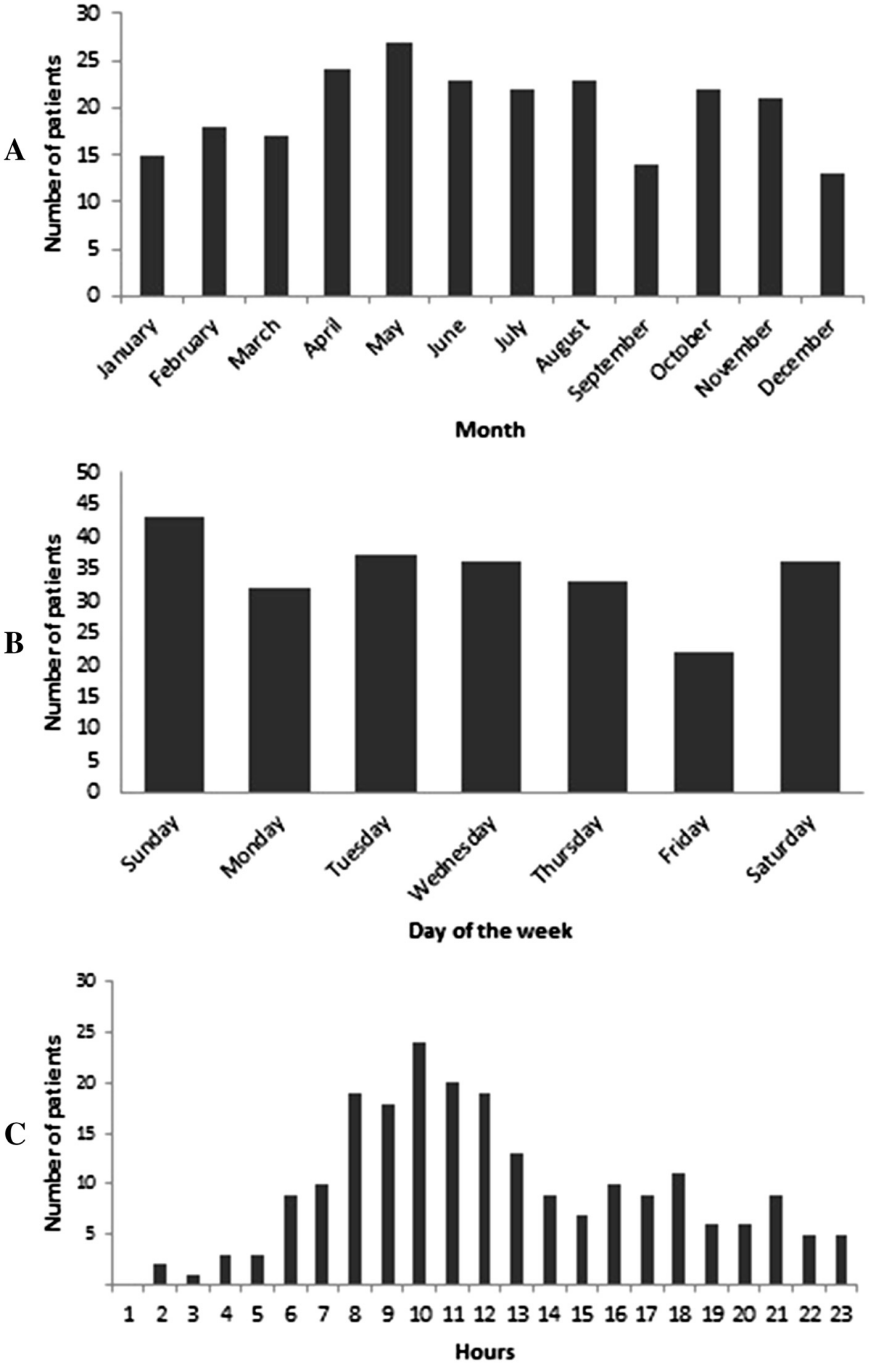
Distribution of hospitalized spinal injury patients by month of the year (**a**) day of the week (**b**) and hours of the day (**c**)

The estimated annual incidence of hospitalized spinal injuries in Al Ain City was 17.4/100,000 persons per year. Most of injuries (119/239) occurred during the months of April-August (Fig. 2a). Sunday had the highest incidence of spine injuries (18 %; *n* = 43) and Friday the lowest (9 %; *n* = 22) (Fig. 2b). More than half of the injuries (113/218) occurred during the morning time (8 a.m. -1 p.m.) (Fig. 2c). The mechanism of injury and its location did not affect the time of injury.

The most common associated injured anatomical regions were the chest (23 %; *n* = 56) with highest AIS, followed by upper extremity (22 %; *n* = 52) and lower extremity (17 %; *n* = 40) (Table 4). Spinal fractures were more common at the lumbar region (57 %; *n* = 136). Twenty nine patients (12 %) had more than one level injured. Eleven patients (5 %) sustained paraplegia, most were associated with lumbar fractures (4/11) (Table 5).

**Table 4:** Associated injured body regions of hospitalized spinal-injured patients, Al Ain, United Arab Emirates, 2003–2006 (*n* = 239)

Region	Number	%	AIS^a^
Head and Neck	33	13.8	2 (1–4)
Face	20	8.4	1 (1–2)
Chest	56	23.4	3 (1–4)
Abdomen	15	6.3	2 (1–3)
Upper extremity	52	21.7	2 (1–3)
Lower extremity	40	16.7	2 (1–3)
External	4	1.67	1 (1–1)

^a^ Data presented as numbers (%) and median (range)

**Table 5:** Distribution of anatomical regions of spinal fractures

Region	Number	%	Paraplegia	%
Cervical alone	28	11.7	0	
Thoracic alone	46	19.2	2	0.8
Lumbar alone	136	57	4	1.7
Cervical and thoracic	7	3	2	0.8
Cervical and lumbar	3	1.2	0	
Thoracic and lumbar	19	7.9	3	1.3
Total	239	100	11	4.6

UAE nationals were significantly more admitted to the ICU compared with non-nationals (*p* = 0.044), and had a shorter hospital stay (*p* = 0.005). There was a strong trend for statistical difference in GCS (*p* = 0.06) and ISS (*p* = 0.06) between UAE and non-UAE nationals. UAE nationals had more severe injuries compared with non UAE nationals. The mean (SD) total hospital stay was 11.4 (11.96) days. Non-nationals were hospitalized longer than UAE nationals (*p* = 0.005) because they had a higher percentage of paraplegia (Table 2). The median (range) ISS of patients was 5 (1–38). Patients with traffic-related spine injury had higher ISS compared with falls from high or same level (*p* < 0.0001) (Table 3). Five patients died (2.1 %), all in traffic collisions.

## Discussion

Our study has shown that men in the productive age, majority from the Indian subcontinent, are at the highest risk for spinal injuries at work in the UAE. National females were more injured at home by falling at same level, while national males were more injured in traffic collisions. Patients injured in traffic had more severe injuries, were more admitted to the ICU, while patients injured by fall from height had longer hospital stay.

The estimated incidence of spinal injuries in our study (174/million population) was much higher than those reported from other countries [18-20]. The mean age of our patients (37.5 years) was similar to other countries, such as Italy, United States and Pakistan [1,3,5], but lower than Japan and China [21,22] because of the different injury risk in the young UAE population. Patients with traffic spine injuries and fall from height in our study were younger compared with those with fall at the same level.

Similar to others [1,8,23], majority of our injured patients were males. The male:female ratio (9:1) was much higher compared with other countries [1,5,23,24]. The overall male:female ratio in UAE is 2:1 due to the large number of expatriate workers [9].

Traffic-related spine injury was the most common mechanism of injury and occurred in young males in our study, similar to other developed countries [5,8,19,25]. Economic growth and increasing use of motor vehicles with improving road infrastructure in the UAE have been followed by increasing rates of traffic injuries. Restraint use is low and enforcement of traffic safety regulations is not appropriate [10].

Fall from height is the most common cause of spine injury in developing countries [1]. It was the second leading cause in our study. Many expatriate workers from the Indian subcontinent are injured in the construction industry [26], which was regarded as the most hazardous industry [27]. Immigrant workers often lack safety equipment and safety education in their own language [10].

We identified a daily time peak during the morning hours (8 a.m.–1 p.m.). A study on occupational injuries from Canada reported a daily peak at 11 a.m. possibly due to sleep deprivation [28]. Falls occurred most often on Sundays, first working day in the week in the UAE and less often on Fridays, which is a weekend. The highest monthly incidence of spine injuries in our study was during spring and summer. It is possible that the high outdoor temperatures, which can reach up to 50 °C in the summer, can decrease the vigilance among our population both in the traffic and at work. A study on occupational injuries from Canada [28] reported the highest peak of injuries in August. Spine injuries caused by fall from height occur more at work. A study from Qatar [26] reported that falling from height at construction sites was common with significant effects on the health care system.

Females in our study were more injured at home by falling at the same level. The age of the patients who fell at the same level was higher compared with other mechanisms. As described elsewhere, falls among elderly can cause serious injuries and death [29]. Hazards in physical environment at home are importact risk factors for falls [30]. We did not record any sport-related spine injury in our study, possibly because high risk sport activities as diving, skiing or gymnastics are not common in Al Ain City.

Similar to others, the most common region that had spinal fractures in our study was the lumbar region [8]. Cervical spinal fractures are more common in patients injured in traffic, while lumbar spinal fractures are more common in falls [8,25]. The transition between the cervical and the thoracic spine has a weak muscular support and is more prone to the acceleration/deceleration impact force during a traffic crash [31]. On the other hand, thoracolumbar junction has a defined muscular structure protecting against distraction forces, but more prone to compression fractures, due to the high pressure on the vertebral body [31]. There is an observed increase in the proportion of complete lumbosacral spine cord injuries because of the progressive increase of falls [23]. It is possible that, with improved traffic safety and increased age of the population, the importance of fall prevention, including prophylaxis of osteoporosis, will increase.

Mortality in our study (2.1 %) was lower than those reported from China (3.4 %) [32], Canada (4 %) [23] or Australia (5.2 %) [24]. All patients who died in our study were injured in traffic collisions. Spinal fractures in road traffic collisions had a higher mortality when compared with falls [25].

The modern traffic design with 2–3 highway lanes in one direction inside Al Ain City with many roundabouts is a high risk for rollover traffic crashes of popular sport utility vehicles leading to the ejection and spine injury of the unrestrained occupants [15]. Seat belt compliance is low in the UAE [15,33]. Proper restraint use may reduce the risk of spine injury and fatality during traffic crashes [34]. The most difficult challenge in the UAE is to change the behavior of the road users [10]. Comprehensive restraint legislation with primary enforcement and culturally appropriate education is a necessity [10].

The high incidence of occupational injuries in the UAE is caused by the large recruitment of workers, especially from Indian subcontinent, and lack of appropriate implementation of safety precautions [10,35]. Occupational setting, such as high construction sites or date palm farms posess a high risk for falls from heights in the UAE. Monitoring of occupational injuries with adequate safety inspections and training is important not only for major employers, but also for smaller entities and farms [10].

Home injury prevention is lacking in the UAE. Due to the hot climate, homes are often built with hard surfaces as marble or tiles. These surfaces do not absorb high impact during falls which increases the risk for injury. Ceilings are high so as to improve cooling. Activities, such as exchanging the electrical bulbs or hanging curtains demand using high ladders and possess a serious fall risk. Popular small carpets at homes without antislippery rubber mat are risk for fall in the elderly in our community. A proper evidence-based architectural design can prevent falls [36].

There are certain limitations in our study. Our study included only patients who were admitted to the hospital for more than 24 h and those who died in the Emergency Department. Patients with more severe spine injuries may have died before arriving to the hospital. Furthermore, our study was based in Al Ain City with less construction sites and lower buildings than Abu Dhabi or Dubai cities. All of this may limit the generalizability of our results for the whole UAE.

It is worthy to note that our data represent the period before 2007 which may not exactly reflect the recent situation. These data were retrieved from Al Ain Hospital Trauma Registry which was the only available trauma registry in our country. It was a specific time limited research project supported by the UAE University. Nevertheless, we think that risk factors for spine injuries in our city did not change since then.

Furthermore, our study is an epidemiological study and not a clinical study. Accordingly we did not stratify our patients by the spinal surgical type and technique [37]. Nevertheless, it is important to highlight that injury prevention is an important integral part of the duties of trauma surgeons. This should include defining injury risk factors, studying the effects of interventional studies on injury prevention, and support health-policy reform through proper research [38-40].

## Conclusions

Traffic injuries and falls were the leading causes for spinal injury is the UAE. Expatriate males are at high risk for fall from height, UAE national males from traffic, and females for falls at the same level at homes. Prevention should focus on traffic and home injuries for UAE nationals and occupational safety for expatriate workers.
